# Correlation between NFκB Signaling and Na^+^, K^+^‑ATPase Inhibition in Vincristine-Induced Emotional and Cognitive Comorbidities in Mice: Neuroprotective Potential of 4‑PSQ

**DOI:** 10.1021/acsomega.5c07423

**Published:** 2026-02-09

**Authors:** Ketlyn Pereira da Motta, Carolina Cristóvão Martins, Vanessa Macedo Esteves da Rocha, Ingrid Cardoso Oliveira, Diego Alves, Larissa Daniele Bobermin, André Quincozes-Santos, Ethel Antunes Wilhelm

**Affiliations:** † Graduate Program in Biochemistry and Bioprospecting, Preclinical and Translational Research Group on Pain and Chronic Diseases, CCQFA, 37902 Federal University of Pelotas (UFPel), P.O., Box 354, 96010-900 Pelotas, Rio Grande do Sul, Brazil; ‡ Graduate Program in Chemistry, Clean Organic Synthesis Laboratory, CCQFA, Federal University of Pelotas (UFPel), P.O., Box 354 96010-900 Pelotas, Rio Grande do Sul, Brazil; § Graduate Program in Biological Sciences: Biochemistry, Institute of Basic Health Sciences, 28124 Federal University of Rio Grande do Sul (UFRGS), 90035-003 Porto Alegre, Rio Grande do Sul, Brazil

## Abstract

Vincristine (VCR), a widely used chemotherapeutic agent, is associated with persistent neurotoxic effects including psychiatric and cognitive impairments in cancer survivors. In contrast, 7-chloro-4-(phenylselanyl)­quinoline (4-PSQ) is a synthetic compound with potential neuroprotective effects. This study investigated the neurobehavioral and molecular effects of VCR in male and female Swiss mice, with a focus on sex-specific outcomes and the neuroprotective efficacy of 4-PSQ. Mice received daily intraperitoneal injections of VCR (0.1 mg/kg) or vehicle (0.9% saline solution) for 5 days, followed by daily oral administration of 4-PSQ (1 mg/kg) or vehicle (canola oil) from days 7 to 16. Behavioral assessments for depressive- and anxiety-like responses and cognitive performance were conducted, followed by biochemical analyses of the cerebral cortex and spinal cord. VCR significantly increased nuclear factor kappa B (NFκB) expression and inhibited Na^+^,K^+^-ATPase activity in both sexes while selectively upregulating protein-21 (protein 21 (p21)) mRNA in the cerebral cortex of females. These molecular alterations correlated with behavioral impairments, as shown by a robust pattern of sex-specific associations: in males, behavioral changes were strongly correlated with Na^+^,K^+^-ATPase inhibition, while in females, increased NFκB expression was more strongly associated with emotional and cognitive deficits. Notably, p21 overexpression has emerged as a unique marker of neurotoxicity in females. Treatment with 4-PSQ attenuated molecular and behavioral alterations in both sexes, supporting its neuroprotective efficacy. By establishing specific correlations between biochemical and behavioral parameters, this study provides mechanistic insights into VCR-induced neurotoxicity. It proposes 4-PSQ as a promising therapeutic candidate for preventing or reversing chemotherapy-related cognitive and emotional dysfunctions.

## Graphical abstract

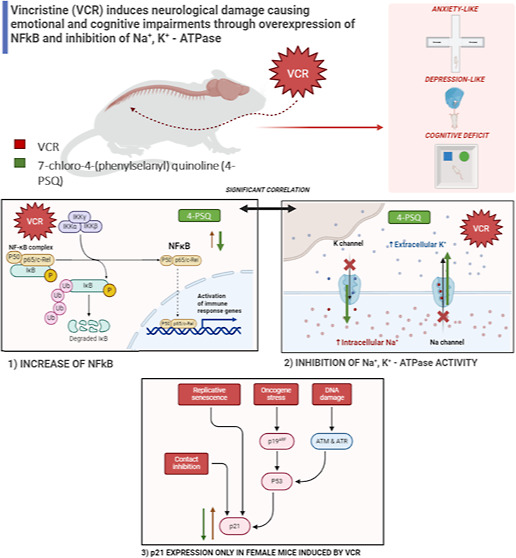

## 1 Introduction

Vincristine (VCR) is a widely used chemotherapy agent in oncology, effectively suppressing tumor proliferation across various age groups.^1–3^ However, its usage is associated with neurotoxicity, inducing mechanisms recently described as “chemo-brain”.^4,5^ VCR-triggered events can compromise neurological functions, potentially leading to cognitive and emotional dysfunctions.^3,6–9^ The interconnected pathways disrupted by VCR suggest a neurochemical mechanism that activates concurrent cascades and disrupts normal neural functionality.

Nuclear factor kappa B (NFκB) regulates essential biochemical pathways in healthy conditions.^10^ Its activation is linked to inflammation, with changes in NFκB expression causing damage to mitochondria, DNA, and the cytoplasm.^10,11^ Evidence indicates that NFκB might act as a regulator in mitochondria, influencing the balance between cytoplasmic glycolysis and mitochondrial respiration in normal cells. In the cytoplasm, NFκB can trigger cell apoptosis by releasing cytochrome C, initiating caspase cascades.^10^ In the nucleus, NFκB can enhance the expression of pro-inflammatory proteins. Although VCR is known to hyperactivate NFκB, its association with psychiatric and cognitive disorders remains underexplored.^12,13^

Conversely, the enzyme Na^+^, K^+^-ATPase is highly involved in axonal depolarization processes and neuronal signaling.^14,15^ Inhibition of Na^+^, K^+^-ATPase can be linked to depressive and anxious behaviors by preventing the release of neurotransmitters associated with well-being.^16–18^ The relationship between Na^+^, K^+^-ATPase activity inhibition, and VCR effects is also unexplored.

Therefore, identifying axonal impairment altered by VCR, its correlation with Na^+^, K^+^-ATPase inhibition, and linking this inhibition to NFκB hyperactivation could represent a neurobiological mechanism that fosters neuroinflammation, cell apoptosis, and consequent cognitive deficits and emotional disorders. Furthermore, considering cellular damage, the role of protein-21 (p21) as a crucial element in cellular health maintenance might indicate another pathway modulated by VCR. It is important to highlight that the role of p21 has already been described as it is hyperexpressed by VCR in situations of Janus Kinase 2 inactivation and medulloblastoma cells. However, the isolated modulation of p21 expression by VCR is unknown.^19,20^ Therefore, given the neurotoxic potential provided by VCR, therapeutic alternatives that can minimize neurological effects are necessary.

7-Chloro-4-(phenylselanyl) quinoline (4-PSQ) exhibits broad therapeutic potential, particularly in pathologies involving neurological impairments such as Alzheimer’s, anxiety, Parkinson’s disease, cognitive decline, and chemotherapy-associated comorbidities in animal models.^18,21–27^ Its pharmacological properties, including antioxidant and anti-inflammatory capacities, neuroplasticity promotion, and modulation of glutamatergic, GABAergic, nitrergic, and serotonergic pathways, are noteworthy.^23,28,29^ Specifically, its serotonergic modulation through 5-HT_1A/1B_ and 5-HT_2A/2C_ receptors significantly alleviates anxiety-like and depression-like behaviors.^28^ Furthermore, 4-PSQ increases the expression of the cell adhesion molecule (NCAM), favoring neuroplasticity and learning and reversing cognitive problems.^24^

Considering the neurological impairment caused by VCR via pathways associated with neuroinflammation and oxidative damage and the neuroprotective potential of 4-PSQ reported by several studies, we hypothesize that VCR induces neurochemical changes correlated with emotional and cognitive dysfunction. We propose that 4-PSQ, a multitarget molecule, has therapeutic potential to counteract the neurotoxic effects of VCR. Therefore, this study aimed to investigate VCR-induced neurotoxicity manifested through emotional and cognitive impairments, focusing on pathways involving NFκB, Na^+^, K^+^-ATPase, and p21 and evaluating the therapeutic efficacy of 4-PSQ.

## 2 Results

### 2.1 Behavior Tests

#### 2.1.1 Locomotor and Exploratory Abilities

As demonstrated in Figure 1, no alterations were observed in the animals’ locomotor and exploratory capacities. Administration of VCR, 4-PSQ, or the combination of VCR + 4-PSQ did not change the number of crossings of male (Figure 1a) and female (Figure 1c) mice, nor did it alter the number of rearings of male (Figure 1b) and female (Figure 1d) mice.

**Figure 1:**
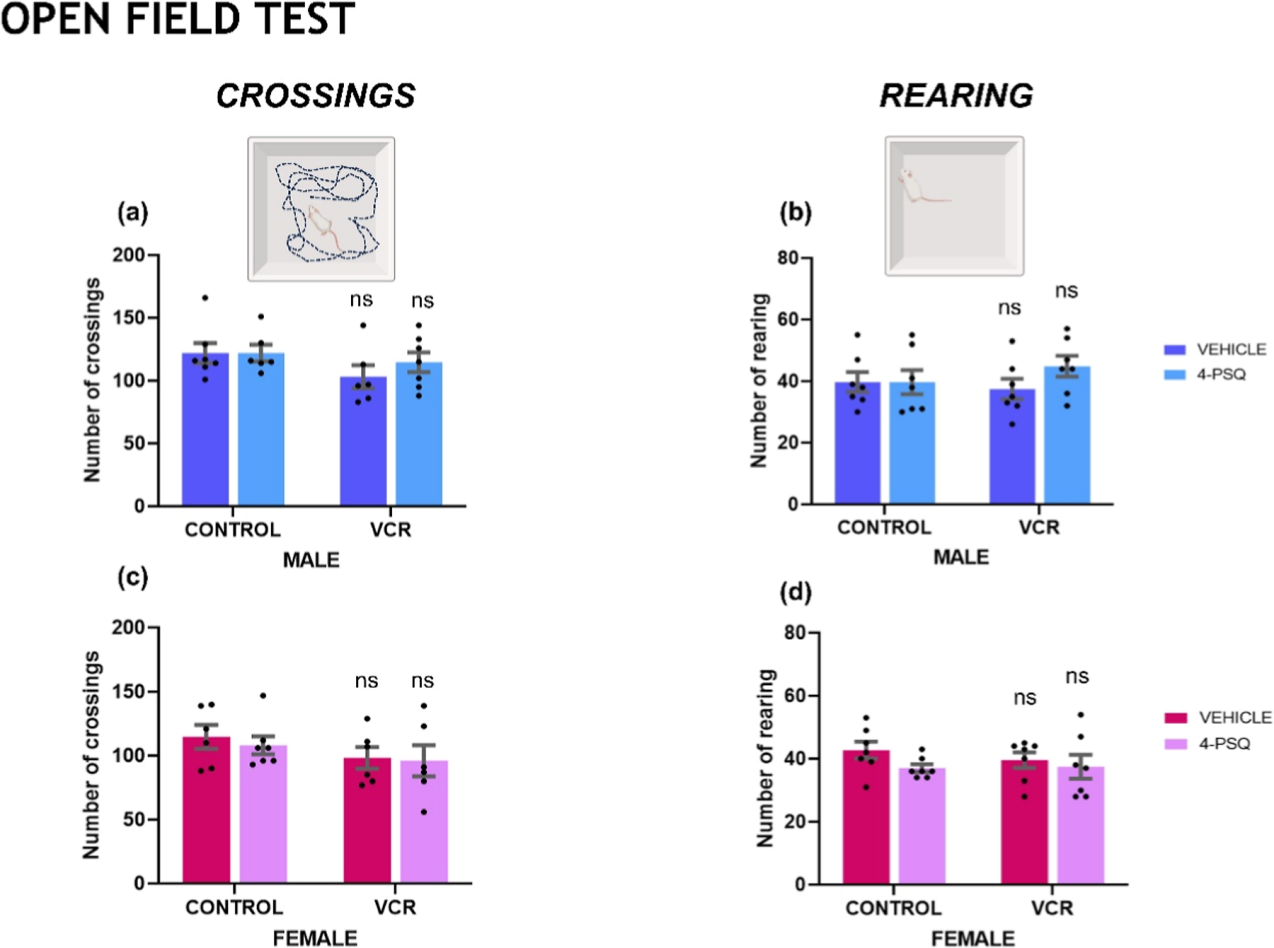
Effect of 7-chloro-4-(phenylselanyl) quinoline (4-PSQ) (1 mg kg^–1^, p.o.) and vincristine (VCR) (0.1 mg kg^–1^, i.p.) on the number of crossings of male (a) and female (c) and the number of rearings of male (b) and female (d) mice. Each column represents the mean ± standard error of the mean (S.E.M) of 7 animals per group. Ns indicated nonsignificant differences. Two-way ANOVA followed by Tukey’s test was used. The schematic illustration was created using BioRender.com.

#### 2.1.2 Investigation of Memory and Cognition

Impairments in the short term (STM) and long-term (LTM) can characterize cognitive deficits. Damage to the central nervous system (CNS) and exposure to neurotoxic drugs such as VCR can hinder cognitive function. Training sessions were conducted with male (Figure 2a) and female (Figure 2d) mice to familiarize the animals with the testing apparatus. No significant differences were observed between the groups during training for the object recognition task. VCR induced a decreased exploratory preference in the STM in male (Figure 2b; 55%) and female (Figure 2e; 48%) mice. Furthermore, LTM was compromised by VCR in male (Figure 2c; 42%) and female (Figure 2f; 48%) mice.

**Figure 2:**
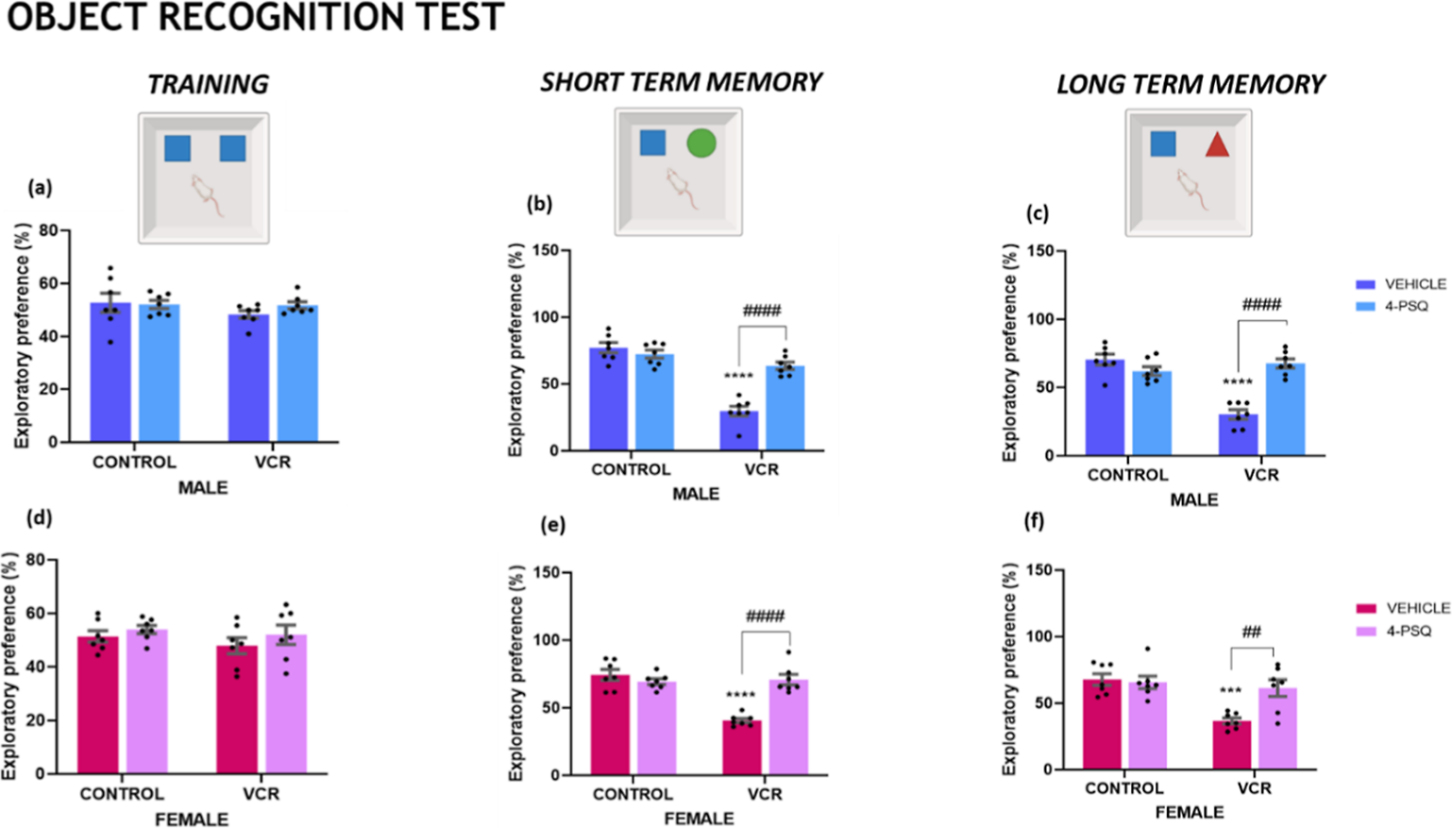
Effect of 7-chloro-4-(phenylselanyl) quinoline (4-PSQ) (1 mg kg^–1^, p.o.) and vincristine (VCR) (0.1 mg kg^–1^, i.p.) on training of male (a) and female (d) mice; in the short term memory of male (b) and female (e) mice and the long-term memory of male (c) and female (f) mice in the object recognition task. Each column represents the mean ± S.E.M of 7 animals per group. (***) *P* < 0.001 and (****) *P* < 0.0001 denote significance levels when compared with the control group; (##) *P* < 0.01 and (####) *P* < 0.0001 denote significance levels compared with the VCR groups. Two-way ANOVA followed by Tukey’s test was used. The schematic illustration was created using BioRender.com.

Therefore, treatment with the compound 4-PSQ restored the cognitive abilities impaired by VCR. The compound’s therapeutic potential on the nervous system proved effective in reinstating STM and LTM in male and female mice subjected to VCR.

#### 2.1.3 Assessment of Depressive-like Behavior

Painful conditions are often linked to the onset of depression. In the tail suspension test, we examined the immobility response to a stressful situation (Figure 3). VCR induced depressive-like behavior in male mice, indicated by a reduced latency time to immobility (Figure 3a; 70%) and an increase in overall immobility (Figure 3b; 97%). Similarly, female mice exposed to VCR exhibited depressive-like behavior, evident in reduced latency time to immobility (Figure 3c; 53%) and increased general immobility (Figure 3d; 159%).

**Figure 3:**
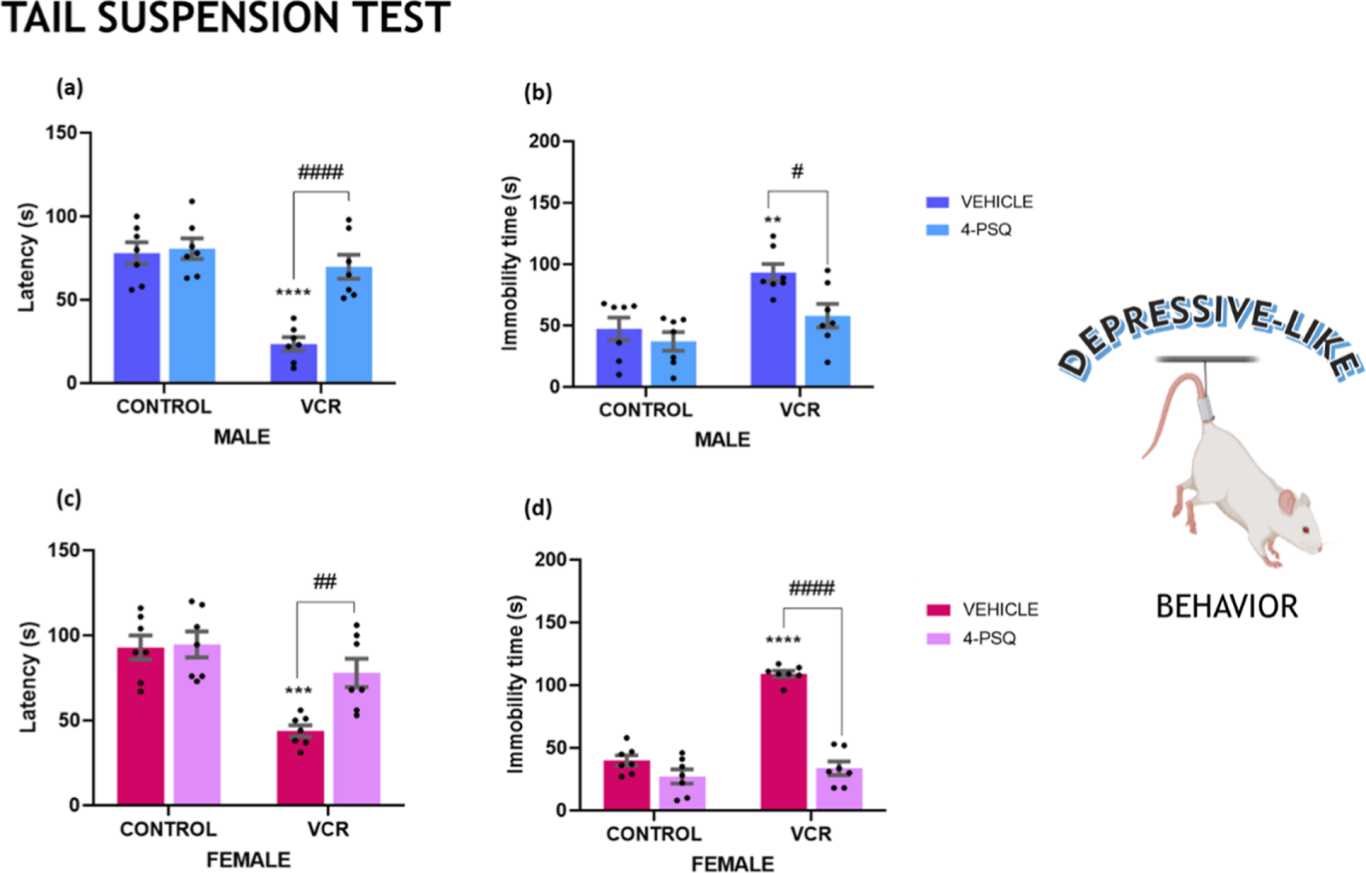
Effect of 7-chloro-4-(phenylselanyl) quinoline (4-PSQ) (1 mg kg^–1^, p.o.) and vincristine (VCR) (0.1 mg kg^–1^, i.p.) on male (a) and female (c) latency and immobility of male (b) and female (d) in the tail suspension test. Each column represents the mean ± S.E.M of 7 animals per group. (**) *P* < 0.01; (***) *P* < 0.001 and (****) *P* < 0.0001 denote significance levels when compared with the control group; (#) *P* < 0.05; (##) *P* < 0.01 and (####) *P* < 0.0001 denote significance levels compared with the VCR groups. Two-way ANOVA followed by Tukey’s test was used. The schematic illustration was created using BioRender.com.

Typically, antidepressant medications demonstrate the ability to normalize changes in this test. 4-PSQ normalized depressive-like behavior in both male and female mice subjected to VCR. The antidepressant effect of 4-PSQ restored latency and general immobility in both genders following chemotherapy induction.

#### 2.1.4 Assessment of Anxious-like Behavior

Anxiety-like behavior reflects emotional changes occurring at a psychological level. The elevated plus maze test is a well-established method for assessing rodent anxiety-like behavior. The VCR exposure resulted in emotional disturbances, as demonstrated in the elevated plus maze test conducted by male and female mice (Figure 4). Among male mice, anxiety-like behavior induced by VCR was observed, indicated by the reduction in time spent in the open arms (Figure 4a; 53%), fewer entries into the open arms (Figure 4b; 30%), and a decrease in the number of dives (Figure 4c; 68%). Similarly, female mice exposed to VCR exhibited reduced time spent in the open arms (Figure 4d; 81%), fewer entries into the open arms (Figure 4e; 67%), and a decreased number of dives (Figure 4f; 54%).

**Figure 4:**
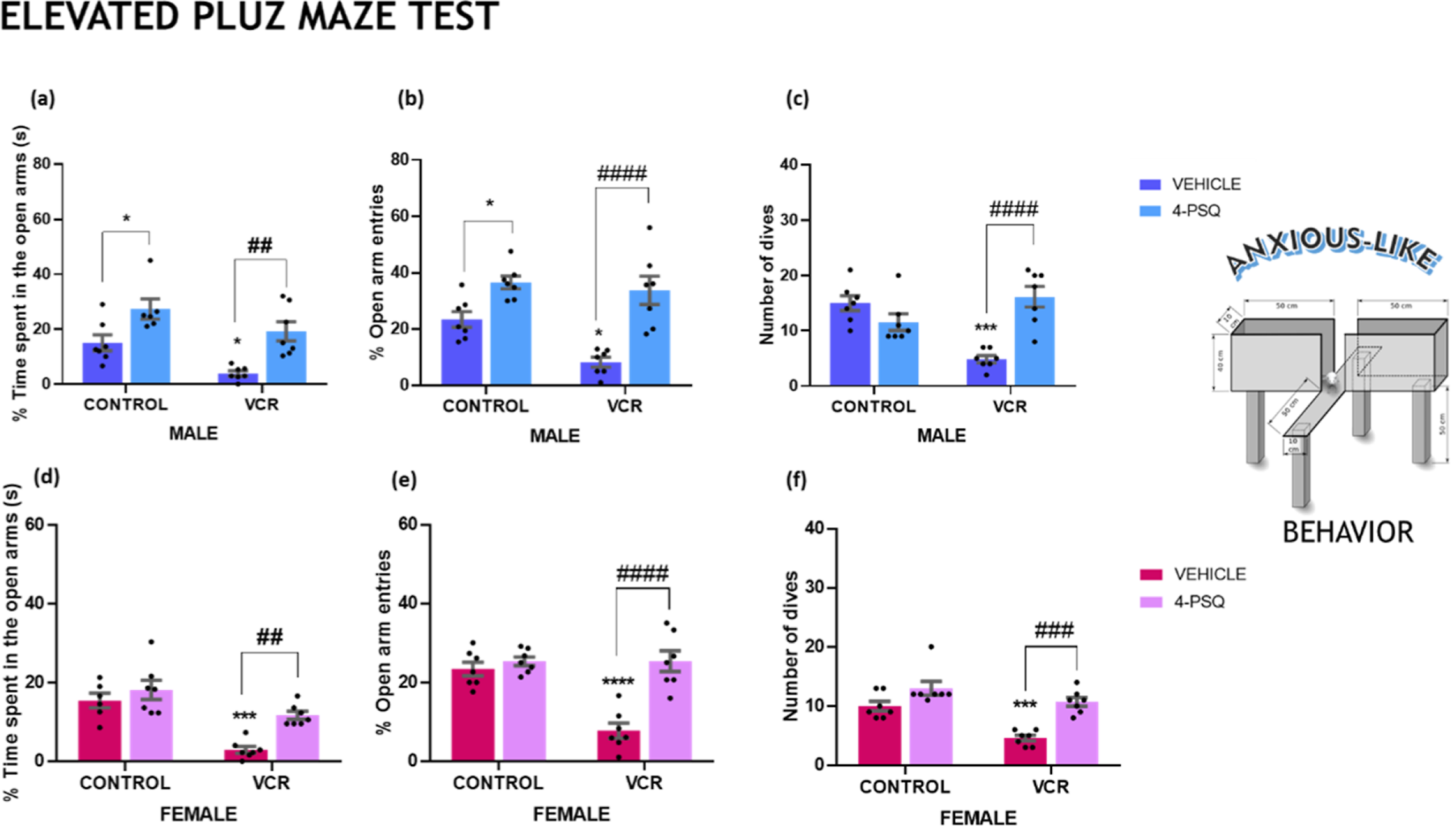
Effect of 7-chloro-4-(phenylselanyl) quinoline (4-PSQ) (1 mg kg^–1^, p.o.) and vincristine (VCR) (0.1 mg kg^–1^, i.p.) on time spent in open arms of male (a) and female (d) mice, entries into the open arms of male (b) and female (e) mice and dives of male (c) and female (f) mice in the elevated plus maze test. Each column represents the mean ± S.E.M of 7 animals per group. (*) *P* < 0.05, (***) *P* < 0.001, and (****); *P* < 0.0001 denotes significance levels when compared with the control group; (##) *P* < 0.01; (###) *P* < 0.001 and (####); *P* < 0.0001 denote significance levels compared with VCR groups. Two-way ANOVA followed by Tukey’s test was used. The schematic illustration was created using BioRender.com.

The therapeutic effect of 4-PSQ restored the reduction in time spent in the open arms, the decrease in entries into the open arms, and the diminished number of dives caused by VCR. The potential anxiolytic effect of 4-PSQ was observed in male and female mice subjected to VCR induction.

### 2.2 Biochemical Assays

#### 2.2.1 Na^+^, K^+^-ATPase Activity

Inhibition of the Na^+^, K^+^ ATPase enzyme activity is crucial in developing neurotoxicity induced by chemotherapy agents. In this context, the inhibitory potential of VCR on Na^+^, K^+^-ATPase activity is illustrated (Figure 5). VCR effectively hindered Na^+^, K^+^-ATPase activity in the cerebral cortex (Figure 5a, 78%), spinal cord (Figure 5b, 76%), and cerebellum (Figure 5c, 56%) of male mice. Similarly, in female mice, VCR exerted inhibitory effects on Na^+^, K^+^-ATPase activity in the cerebral cortex (Figure 5e, 47%), spinal cord (Figure 5f, 46%), cerebellum (Figure 5g, 51%), and hippocampus (Figure 5h, 42%). Notably, no alterations were observed in the Na^+^, K^+^ ATPase activity in the hippocampus of male mice (Figure 5d).

**Figure 5:**
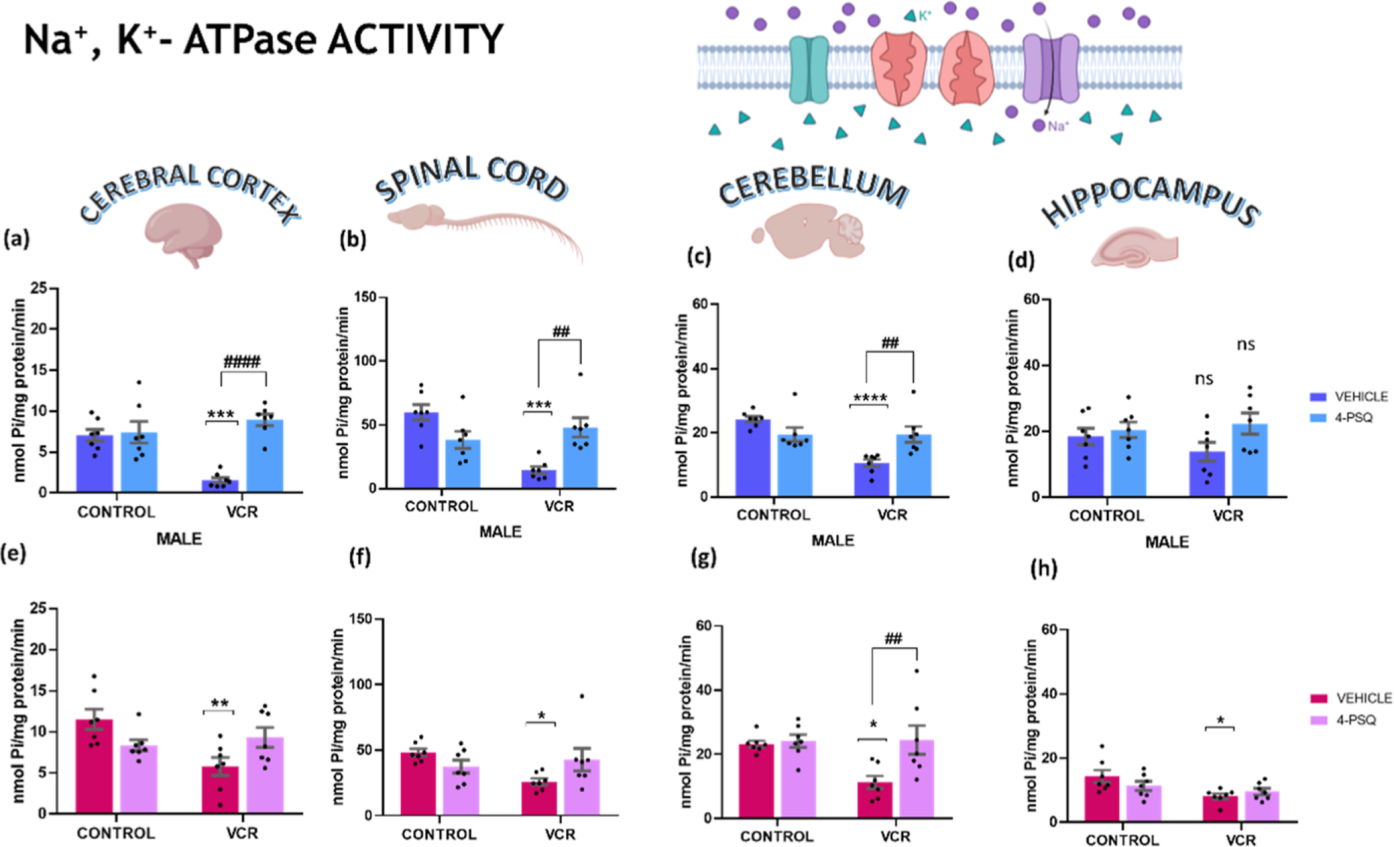
Effect of 7-chloro-4-(phenylselanyl) quinoline (4-PSQ) (1 mg kg^–1^, p.o.) and vincristine (VCR) (0.1 mg kg^–1^, i.p.) on Na^+^, K^+^-ATPase activity. Na^+^, K^+^-ATPase activity in the cerebral cortex of male (a) and female (e); in the spinal cord of male (b) and female (f); in the cerebellum of male (c) and female (g), and in the hippocampus of male (d) and female (h) mice. Each column represents the mean ± S.E.M of 7 animals per group. (*) *P* < 0.05, (**) *P* < 0.01, (***) *P* < 0.001, and (****) *P* < 0.0001 denote significance levels when compared with the control group. (##) *P* < 0.01 and (####) *P* < 0.0001 denote significance levels compared with the VCR group. Ns indicates nonsignificant difference*s*. Two-way ANOVA followed by Tukey’s test was used. The schematic illustration was created using BioRender.com.

Conversely, 4-PSQ effectively counteracted the inhibition of Na^+^, K^+^ ATPase induced by VCR in the cerebral cortex, spinal cord, and cerebellum of male mice. In females, 4-PSQ normalized Na^+^, K^+^ ATPase activity solely in the cerebellum.

#### 2.2.2 p21 mRNA Expression Levels

The p21, when altered, induces a phenotypic response related to changes in the cell cycle and can cause cellular damage from this deregulation. The gene expression of p21 is demonstrated in Figure 6. The experimental data indicated that VCR increased the expression of p21 only in the cerebral cortex of female mice (Figure 6b, 50%). When VCR induced an increase in p21 expression, compound 4-PSQ normalized it. VCR and 4-PSQ did not affect p21 expression in the cerebral cortex and spinal cord of male mice nor in the spinal cord of female mice (Figure 6a,c,d).

**Figure 6:**
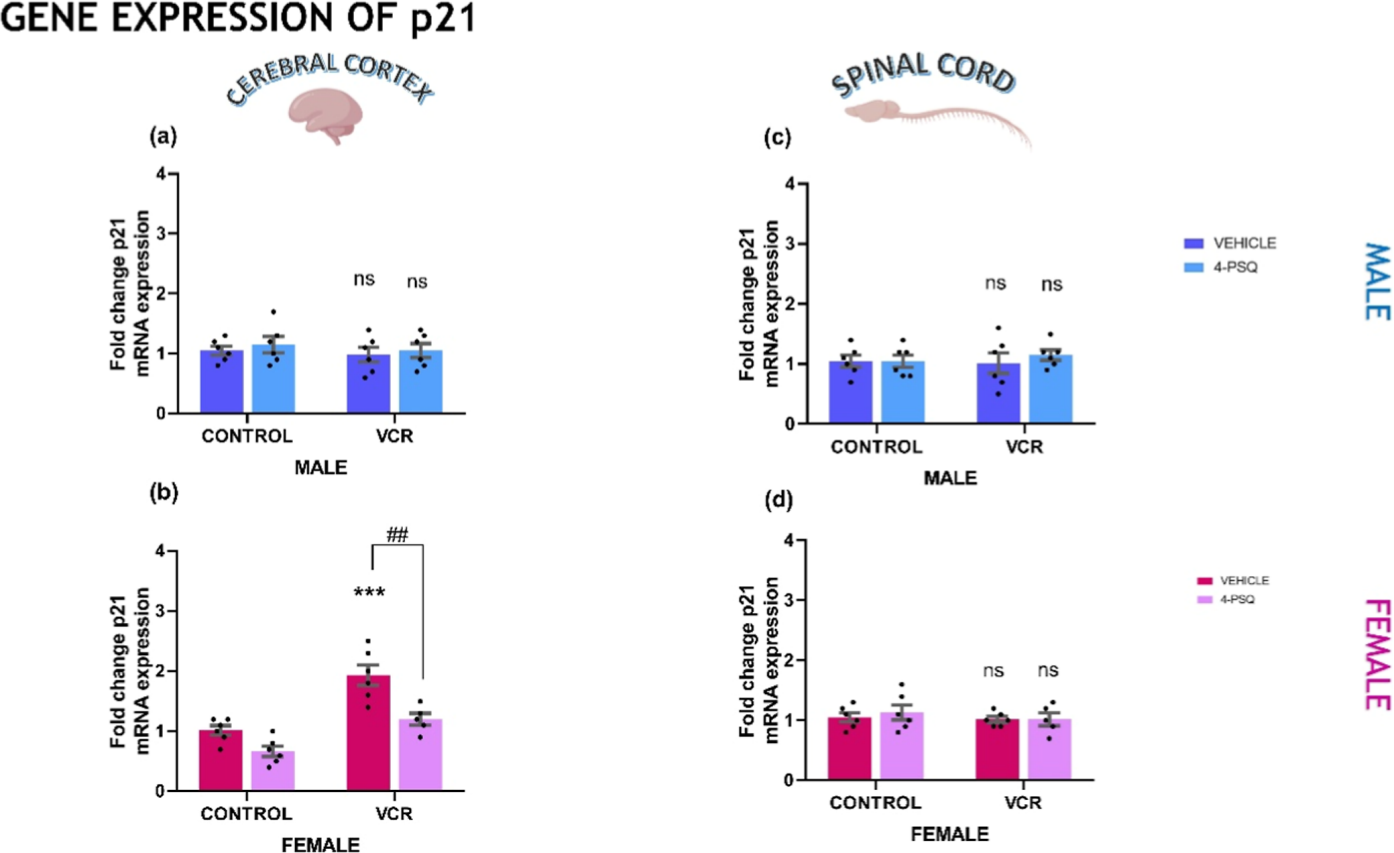
Effect of 7-chloro-4-(phenylselanyl) quinoline (4-PSQ) (1 mg kg^–1^, p.o.) and vincristine (VCR) (0.1 mg kg^–1^, i.p.) on protein 21 (p21) mRNA expression levels. The p21 gene expression in the cerebral cortex of male (a) and female (b); and in the spinal cord of male (c) and female (d) mice. Each column represents the mean ± S.E.M of 6 animals per group. (***) *P* < 0.001 denotes significance levels compared with the control group. (##) *P* < 0.01 denotes significance levels compared with the VCR group. Ns indicated nonsignificant difference*s*. Two-way ANOVA followed by Tukey’s test was used. The schematic illustration was created using BioRender.com.

#### 2.2.3 NFκB mRNA Expression Levels

Signaling through NFκB is associated with neuroinflammation, activation of apoptosis pathways, impaired memory, and emotional damage. The NFκB gene expression observed in this study is demonstrated in Figure 7. Our data showed that VCR administration led to increased NFκB gene expression in the cerebral cortex of male (Figure 7a; 36%) and female (Figure 7b; 135%) mice and in the spinal cord of male (Figure 7c; 57%) and female (Figure 7d; 64%) mice. 4-PSQ regulated this gene expression, normalizing NFκB to control levels in the cerebral cortex of males and the spinal cord of male and female mice.

**Figure 7:**
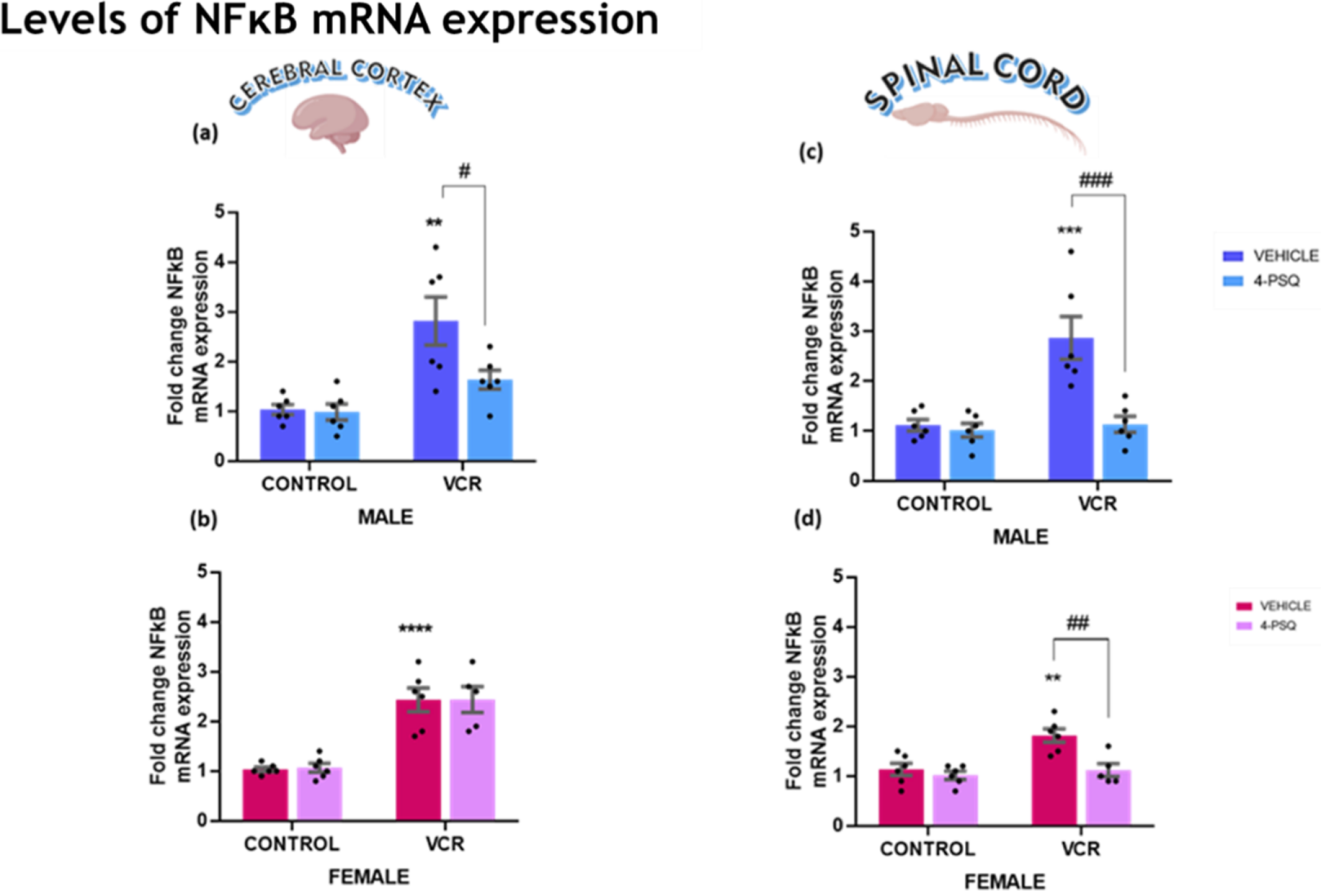
Effect of 7-chloro-4-(phenylselanyl) quinoline (4-PSQ) (1 mg kg^–1^, p.o.) and vincristine (VCR) (0.1 mg kg^–1^, i.p.) on levels of mRNA expression of nuclear factor kappa B (NFκB). NFκB gene expression in the cerebral cortex of male (a) and female (b), and in the spinal cord of male (c) and female (d) mice. Each column represents the mean ± S.E.M of 6 animals per group. (**) *P* < 0.01, (***) *P* < 0.001, and (****) *P* < 0.0001 denote significance levels compared with the control group. (#) *P* < 0.05, (##) *P* < 0.01, and (###) *P* < 0.001 denote significance levels compared with the VCR groups. Two-way ANOVA followed by Tukey’s test was used. The schematic illustration was created using BioRender.com.

### 2.3 Correlation between Cerebral Cortex Analysis and Behaviors

The heat graph (Figure 8) demonstrates the correlation between tests and presents the correlation coefficient (*r*) of male (Figure 8a) and female (Figure 8b) mice. Values between 0.7 and *r* ≤ 1 were considered closest to a perfect positive correlation. Values between −1 and *r* ≤ −0.7 were considered closer to a perfect negative correlation.

**Figure 8:**
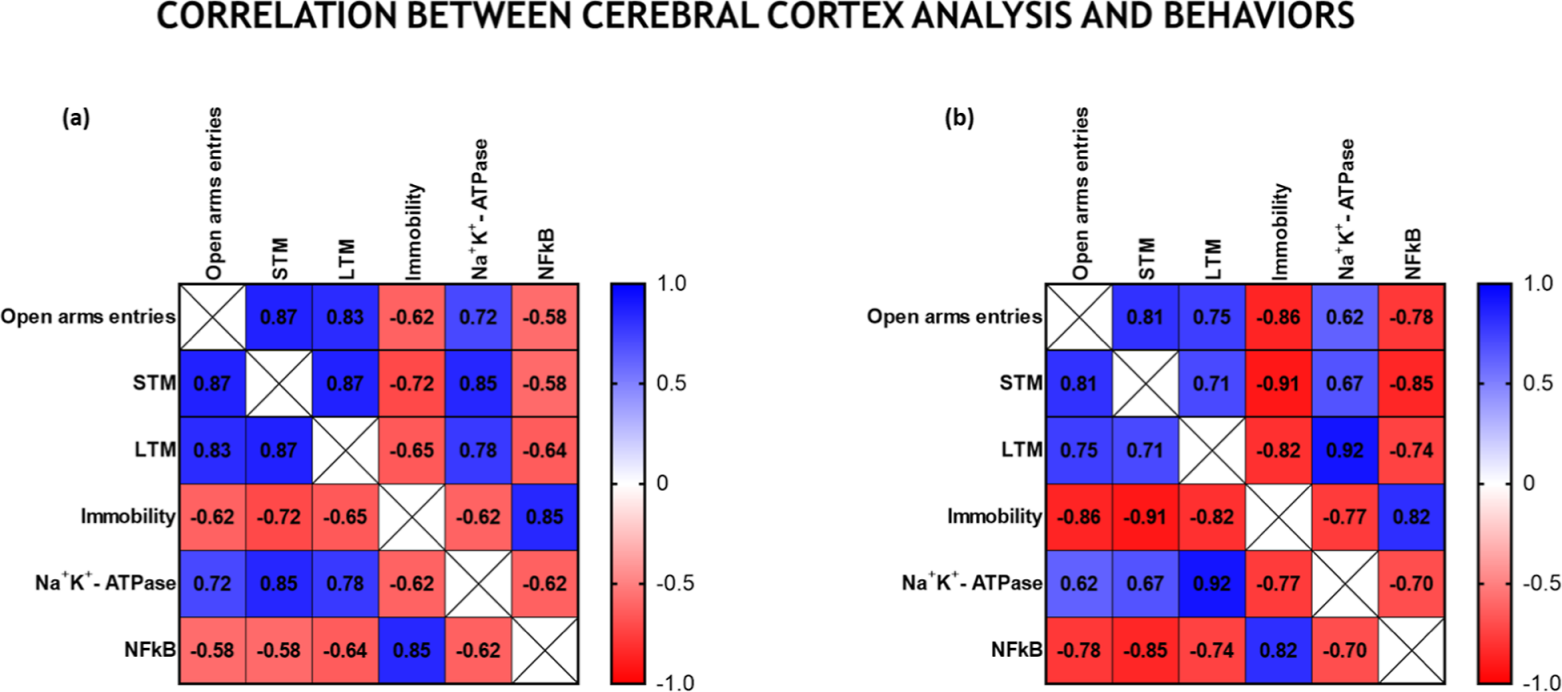
Correlation between emotional and cognitive behaviors with biochemical analyses in the cerebral cortex of male (a) and female (b) mice. Pearson’s correlation coefficient (*r*) was indicated within the quadrants. Values of *r* ≅ 1 indicate a positive correlation between the variables. Values of *r* ≅ −1 indicate a negative correlation between the two variables. Exploratory preference for new objects was used as a parameter collected in the object recognition task to assess STM and LTM memory; the number of entries into the open arms was used as a measure of anxiety-like behavior in the elevated plus maze test, and immobility was used as a parameter in the tail suspension test.

In male mice, there was a positive correlation between the decrease in the exploratory preference of STM and LTM. There was also a positive correlation between the decrease in entries into the open arms and the reduction in exploratory preference (in STM and LTM). At the same time, inhibition of Na^+^, K^+^-ATPase activity showed a positive correlation with the reduction in exploratory preference in STM and LTM. Inhibition of Na^+^, K^+^-ATPase activity also correlated with a reduction in entries into the open arms. The increase in the immobility time correlated positively with increased NFκB mRNA expression. Still, in male mice, there was a negative correlation between decreases in exploratory preference in the STM and increases in the general immobility time (indicating that decreases in preference for the new object are associated with increases in general immobility). Table S1 (Supporting Information) presents P values demonstrating the statistical significance for male mice.

Likewise, in female mice, statistical analysis demonstrated a positive correlation between the decrease in exploratory preference in STM and the decrease in exploratory preference in LTM. The reduction in exploratory preference in STM and LTM also showed a positive correlation with decreased entries into open arms. Inhibition of Na^+^ K^+^-ATPase positively correlates with decreased exploratory preference in LTM. Furthermore, increased NFκB expression was positively correlated with an increased overall immobility time. A negative correlation was observed in the decrease in exploratory preference (both in STM and LTM) with the increase in general immobility; also, there was a negative correlation between the increase in the number of entries into the open arms and the increase in immobility. In parallel, increased expression of NFκB negatively correlated with a decrease in exploratory preference (in LTM and STM) and an increase in the number of entries into open arms.

The correlation coefficient also indicates a greater appeal for a correlation between the inhibition of the enzyme Na^+^, K^+^ ATPase, and behavioral changes in male mice. At the same time, the increase in NFκB expression has a greater correlation with changes that can favor cognitive and emotional impairments in females. Table S2 presents *P* values that demonstrate the statistical significance for female mice.

## 3 Discussion

We observed, for the first time, alterations in NFκB signaling, p21 expression, and Na^+^, K^+^-ATPase activity that are associated with emotional and cognitive changes induced by VCR. Our findings indicate that VCR leads to cognitive and emotional disorders in male and female mice. We hypothesize that VCR-induced inhibition of Na^+^, K^+^-ATPase activity may contribute to changes in neuronal depolarization, which could be related to the observed cognitive and emotional alterations. Additionally, increased NFκB expression suggests the activation of neuroinflammatory and apoptotic pathways in VCR-exposed animals. The exclusive increase in p21 expression in the cerebral cortex of female mice may indicate greater impairment in emotional and cognitive processing, particularly in short-term memory. Notably, the selenium compound 4-PSQ treatment attenuated the cognitive and emotional alterations induced by VCR, suggesting its potential as a neuroprotective candidate against VCR-associated neurotoxicity.

Chemotherapy-related cognitive decline, or “chemo-brain”, affects 17–75% of patients, manifesting as memory impairment and cognitive dysfunction.^4,5^ In our study, VCR impaired short- and long-term memories in male and female mice, corroborating its neurotoxic effects. VCR-induced neurological damage leads to axonal degeneration and dysfunction in astrocytes, microglia, and neurons.^7^ Our data support a potential association between VCR-induced cognitive impairments, reduced Na^+^, K^+^-ATPase activity, and increased NFκB expression. Memory consolidation, dependent on synaptic function, is altered by VCR.^30,31^ Na^+^, K^+^-ATPase, crucial for synaptic functioning, is inhibited by VCR, preventing axonal depolarization, potentially due to reduced ATP affinity under oxidative stress conditions.^32–34^

Our experimental data indicate that the VCR inhibits the Na^+^, K^+^ pump, preventing axonal depolarization, which may be associated with the low affinity of ATP in these cells to provide the nerve impulse. The α and β subunits of Na^+^, K^+^-ATPase comprise around 24 thiols that are extremely sensitive to oxidative stress as they are targets of free radicals, and the VCR may also be interfering with the enzyme’s activity due to oxidative stress.^35,36^

Furthermore, it has been hypothesized that in the cerebral cortex, where Na^+^, K^+^-ATPase activity is inhibited, explicit memory of visual stimuli reestablishes perceptual representations in visual areas.^37^ The object recognition test is carried out with the rodent needing to explore new objects based on the experience of remembering the old object already explored.^38^ Therefore, inhibited Na^+^, K^+^-ATPase activity can prevent the neurochemical perception of memories in the face of visual stimuli.^36^

The hippocampus, essential for spatial memory and the transition of short-term to long-term memory,^39,40^ showed no alteration in Na^+^, K^+^-ATPase activity in male mice. Therefore, the impairment in Na^+^, K^+^ ATPase activity not caused in the hippocampus of male mice indicates that STM is not processed in the long term by the hippocampus through evocation by signal transduction initiated by the Na^+^, K^+^ pump. In female mice, a hypothesis can be raised that VCR causes the inhibition of Na^+^, K^+^-ATPase and thus leads to damage in the transformation of STM through object recognition into LTM. Therefore, we can infer that the exposure of male mice to VCR leads to impairment of memory consolidation through other pathways that are not dependent on Na^+^, K^+^-ATPase. One of the routes that may explain cognitive impairment is the increased level of expression of NFκB in all tissues observed in our study.

Correlation analysis demonstrated that reduced Na^+^, K^+^-ATPase activity in the cerebral cortex is associated with emotional (anxiety- and depression-like) and cognitive impairments. It should be noted that this seems to be a mechanism more associated with damage to the CNS of male mice. In other words, our correlation analysis reinforces this as a mechanism strongly affected by VCR. Male mice may be more susceptible to synaptic changes due to inhibiting the Na^+^, K^+^-ATPase enzyme. The cause of this action may be oxidative stress or it may be directly influenced by the action of VCR by penetrating the blood–brain barrier.^41,42^ However, it is postulated that the inhibition of Na^+^, K^+^-ATPase may be derived from the consolidation of oxidative stress since the increase in NFκB expression may also occur as a result of this.^43,44^

The general activity of Na^+^, K^+^-ATPase is crucial for multiple cellular functions, including maintaining resting membrane potential, regulating cell volume and pH, and driving secondary active transport, all linked to anxiety and depression.^14^ The α2 subunit specifically relates to emotional changes such as fear, anxiety, compulsivity, and hypoactivity.^35^ Excess extracellular K^+^ and glutamate during intense neuronal activity, if not efficiently removed by astrocytes, can lead to widespread depolarization, depressing neuronal firing, and neurotransmitter release, contributing to depressive and anxiety-like behaviors.^45,46^ Therefore, these studies corroborate the contributing role of Na^+^, K^+^ ATPase for the development of anxiety and depression in neurotoxic conditions, such as those induced by VCR.

NFκB signaling is an alert for pathogenic and cellular danger signals, with a potential relationship between neuronal excitability processes and NFκB accumulation.^10,11^ In other words, there may be a relationship between the inhibition of Na^+^, K^+^-ATPase and the increase in NFκB expression. Davis et al.^47^ reported that the inflammatory process, through the increase in nitric oxide (NO), can inhibit nuclear translocation and favor the accumulation of factors such as NFκB. Inhibition of NFκB translocation may reduce DNA binding capacity, which may inhibit nervous system excitability.^48,49^ Our analysis showed an inverse relationship between NFκB and Na^+^, K^+^-ATPase, indicating that increased NFκB expression typically accompanies decreased Na^+^, K^+^-ATPase activity.

The inflammatory process caused by NFκB can initially promote the hyperproduction of pro-inflammatory cytokines, during the activation of inflammatory cascades, which can favor the formation of NO, generating the accumulation of NFκB and impairing the nuclear translocation of this factor.^50^ NFκB accumulation can affect the immune response through changes in T cells, B cells, and dendritic cells.^51^ Furthermore, the accumulation of NFκB can promote abnormal cell survival, favoring uncontrolled cellular nutrition, which needs attention since dysfunctional cells will remain functioning.^10^ This point is in line with the results observed in this study by evaluating the p21 expression. p21 acts as an inhibitor of cyclin-dependent kinases, enzymes responsible for controlling the cell cycle. Since p21 was not altered by VCR in the cerebral cortex of males and in the spinal cord of male and female mice, it is likely that VCR (via NFκB) may not be acting as an inhibitor of CDKs. However, it is noteworthy that in the cerebral cortex of female mice VCR increases p21 expression levels, indicating that NFκB may accumulate more in this tissue and influence neuroinflammation and abnormal cellular maintenance.

NFκB expression was also correlated with depressive- and anxiety-like behaviors, as well as with cognitive impairments induced by VCR exposure.^52,53^ These findings suggest that NFκB signaling may play a significant role in the molecular alterations associated with such behavioral changes.

In the CNS, NFκB regulates learning and memory. The localization of NFκB in synaptosomes suggests a function of NFκB as a retrograde messenger.^11,54^ This mechanism may lead to the loss of axonal transport, already described as a target of the neurotoxic action of VCR.^55^ Therefore, the accentuated expression of NFκB may be associated with cognitive alterations (STM and LTM) and axonal damage observed after VCR exposure.

Concerning emotional damage, it is reported that the dysregulation of NFκB levels may be involved in the depression process. An example of this is the hyperacetylation of NFκB-p65, capable of inducing the positive regulation of the metabotropic glutamate receptor mGlu2, an important target for the action of antidepressant drugs.^56,57^ VCR in this context, through these common pathways, Na^+^, and K^+^-ATPase/NFκB, can lead to emotional and cognitive dysregulation, promoting significant losses in male and female mice.

Regarding the effectiveness of 4-PSQ in reversing cognitive deficits and emotional losses, we can verify some key points in which the molecule may be helping therapeutically. One of the main points possibly linked to the neuroprotective potential of 4-PSQ is its anti-inflammatory property, which has been previously described and can be used to combat the neurotoxic action of VCR.^25,26,28,58^ We infer that 4-PSQ may act as a mediator to combat neuroinflammation, as this mechanism of 4-PSQ has already been reported as one of the main pharmacological targets of the compound, reducing the expression of inflammatory mediators.^23,25^ Furthermore, NFκB promotes the expression of pro-inflammatory cytokines such as interleukins (IL-33, IL-18, IL-1β), which can also be reversed by treatment with 4-PSQ.^26^ This mechanism of anti-inflammatory 4-PSQ can be considered important for the reversal of cognitive impairment and depressive-like and anxiety-like behavior caused by VCR.^16,26^ Furthermore, in a study involving restraint stress, 4-PSQ normalized the phosphatidylinositol-3-kinase (PI3K)/protein kinase B (AKT) pathway, which is activated by neuroinflammatory processes that lead to neuronal dysregulation, impairing neuronal survival and neuroplasticity.^26^

It is important to note that the proposed mechanism underlying the neuroprotective effects of 4-PSQ in this study is hypothetical and based on its previously described antioxidant and anti-inflammatory properties.^59–61^ Although our findings show that 4-PSQ attenuated the behavioral and molecular alterations induced by VCR, the specific molecular pathways involved were not directly assessed in this model. Thus, we infer that the observed beneficial effects may be related to the compound’s known ability to modulate oxidative and inflammatory processes, as reported in earlier studies; however, this hypothesis remains to be experimentally validated.

Neuroplasticity is a function that is directly affected by neurotoxic agents and that impairs memory recall. In this sense, VCR possibly led to losses of neuroplasticity, which may have promoted memory loss. Also, by regulating Na^+^, K^+^-ATPase, 4-PSQ can modulate neuronal excitability and regulate synapses, assisting in neuroplasticity and learning new memories.^16,24^ 4-PSQ regulated Na^+^, K^+^-ATPase in models of anxiety, Alzheimer’s, and peripheral neuropathy caused by oxaliplatin and paclitaxel.^18,23,25–27^

Regarding the comparison of mechanisms between the sexes, our findings demonstrate equal development of psychiatric disorders (anxiety and depression) as well as cognitive deficits between male and female mice. In other words, there was no disparity in susceptibility to developing the emotional and cognitive behaviors assessed. However, we verified p21 as a specific and modified mechanism only in female mice exposed to VCR. Indeed, the relationship between p21 and progesterone, a female hormone, has already been reported.^62^ Progesterone regulates p21-activated kinase 4 (Pak4), which, in pathological situations, can activate inflammatory cascades, suggesting a unique neuroinflammatory mechanism in female mice.^62^

## 4 Conclusions

VCR remains one of the most effective chemotherapeutic agents for the treatment of various malignancies; however, its therapeutic benefits are frequently limited by neurotoxic side effects including peripheral neuropathy, cognitive impairment, and emotional disturbances. Despite its long-standing clinical relevance, the molecular mechanisms underlying these adverse effects remain poorly understood and effective neuroprotective interventions remain lacking.

In this study, we showed that VCR-induced neurotoxicity is associated with increased NFκB expression and reduced Na^+^,K^+^-ATPase activity, supporting a mechanistic interplay between inflammatory signaling and neuronal dysfunction. Notably, treatment with 4-PSQ attenuated both behavioral alterations and molecular disruptions induced by VCR, underscoring its potential as a novel neuroprotective compound.

These findings directly address the urgent need for interventions capable of preventing or mitigating chemotherapy-induced neurological damage without interfering with the antitumor efficacy. Our data contribute to a deeper understanding of the cellular and molecular alterations underlying VCR-induced neurotoxicity and highlight 4-PSQ as a promising therapeutic candidate.

Future investigations should further delineate the specific molecular targets of 4-PSQ and characterize its pharmacokinetic, toxicological, and safety profiles. In addition, advanced delivery strategies, such as polymeric carriers or targeted nanoformulations, may enhance the compound’s stability, bioavailability, and translational potential. These steps are essential for advancing 4-PSQ toward clinical application.

In summary, our findings broaden the current understanding of the mechanisms underlying VCR-induced neurotoxicity and introduce 4-PSQ as an innovative molecule capable of mitigating emotional, cognitive, and neurochemical impairments. This study provides a strong foundation for future formulation development and translational research aimed at improving the quality of life of patients undergoing chemotherapy.

## 5 Materials and Methods

### 5.1 Animals

The experiments used male and female Swiss mice (60 days). The animals were housed with free access to standard chow and water; humidity (20–80%) and temperature (22 ± 2 °C) were controlled with respect to the rodents’ light/dark cycle (12 h/12 h). All experiments were carried out following the Committee for the Care and Use of Experimental Animal Resources guidelines of the Federal University of Pelotas, Brazil (CEEA 4506-2017).

### 5.2 Drugs

The compound 4-PSQ (Figure 9) was synthesized and characterized by the Clean Organic Synthesis Laboratory (LASOL) of the Federal University of Pelotas. The chemical structure was characterized through nuclear magnetic resonance analysis (^1^H and ^13^C). The purity percentage of the compound 4-PSQ was verified by gas chromatography coupled with mass spectrometry (GC–MS), indicating 99.9% purity in the molecule.^63,64^ VCR chemotherapeutic was purchased as sulfate from INTAS Pharmaceuticals (Batch: M2103904) and allocated at 4 °C.

**Figure 9:**
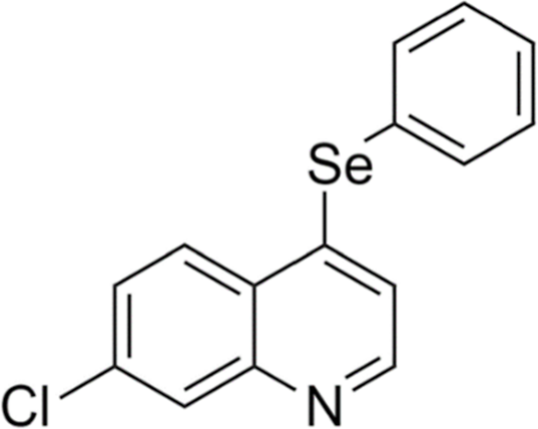
Chemical structure of 7-chloro-4-(phenylselanyl) quinoline (4-PSQ).

Due to its liposoluble property, 4-PSQ was prepared at 1 mg kg^–1^ in canola oil (10 mg mL^–1^; orally with gavage (p.o.)). The VCR was prepared at 0.1 mg/kg^–1^ in 0.9% saline solution (10 mg mL^–1^; intraperitoneal (i.p.)) according to the recommended preparation method.^3^ All other reagents used in this study to perform biochemical techniques were of analytical grade and were obtained from Sigma-Aldrich (St. Louis, MO, USA).

### 5.3 Experimental Design

This study sought to investigate emotional and cognitive changes associated with VCR induction and the therapeutic potential of 4-PSQ. Subsequently, biochemical analyses were carried out to elucidate possible mechanisms related to the development of depressive-like, anxiety-like behavior and cognitive deficits in male and/or female mice.

Male and female Swiss mice were divided into four experimental groups: (I) Control, (II) VCR, (III) 4-PSQ, and (IV) VCR + 4-PSQ. Established protocols designed the experimental design for VCR-induced neurotoxicity.^65–68^ The following section describes how the experimental groups, routes of administration, and exposure schedule were organized:

- Group I—Control: Animals received 0.9% saline solution (10 mL·kg^–1^, i.p.) from Day 1 to Day 5 and canola oil (10 mL·kg^–1^, p.o.) from Day 7 to Day 16.
- Group II—VCR: Animals received VCR (0.1 mg·mL^–1^, i.p.) once daily from Day 1 to Day 5 to induce peripheral neuropathy, followed by canola oil (10 mL·kg^–1^, p.o.) from Day 7 to Day 16.
- Group III—4-PSQ: Animals received 0.9% saline solution (10 mL·kg^–1^, i.p.) from Day 1 to Day 5 and were subsequently treated with 4-PSQ (1 mg·kg^–1^, p.o.) from Day 7 to Day 16.
- Group IV—VCR + 4-PSQ: Animals received VCR (0.1 mg·mL^–1^, i.p.) once daily from Day 1 to Day 5 and were subsequently treated with 4-PSQ (1 mg·kg^–1^, p.o.) from Day 7 to Day 16.

On days 12 and 13, the locomotor and exploratory capacities of the animals were observed, followed by a cognitive assessment in the object recognition test. On day 14, depressive-like behavior was evaluated by the tail suspension test. On day 15, anxiety-like behavior was verified using the elevated plus maze test. Finally, on day 17 of the experimental protocol, all animals were euthanized and brain structures were removed for biochemical analyses. The experimental design is summarized graphically in Figure 10.

**Figure 10:**
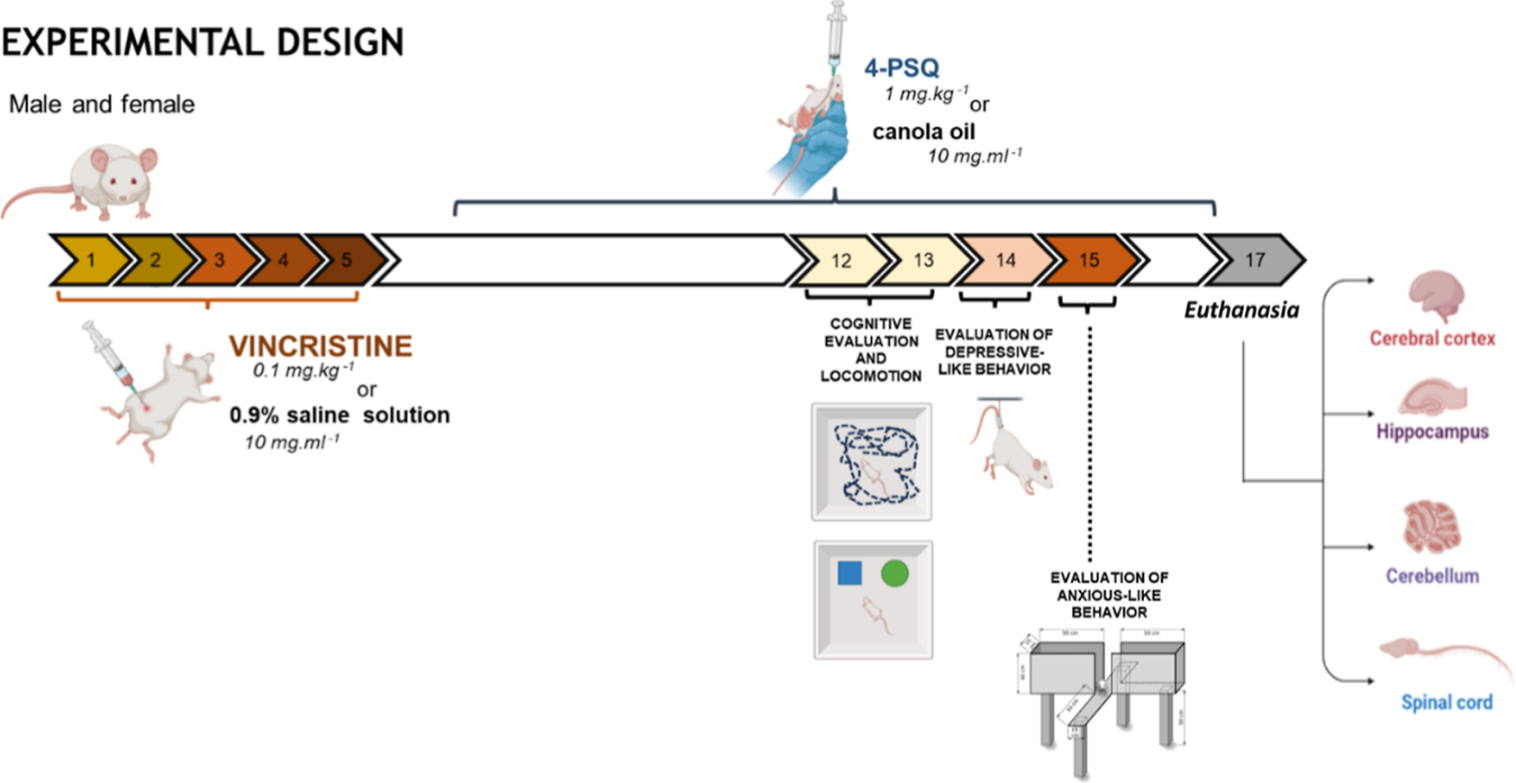
Experimental design scheme. The schematic illustration was created using BioRender.com.

### 5.4 Behavior Tests

#### 5.4.1 Locomotor and Exploratory Abilities

On the 12th day of the experimental protocol, the open field test was conducted following the procedure described by Walsh and Cummins.^69^ The open field apparatus, constructed from plywood, had walls that were 30 cm high. The floor of the open field measured 45 cm in length and 45 cm in width and was divided into 9 squares using masking tape markers (arranged in 3 rows of 3). During the test, each animal was placed at the center of the open field and observed for 4 min to record their locomotion (the number of segments crossed with all four paws) and exploration (the number of times they reared with their hind limbs). After completing the test, the animals were allowed an additional 1 min to adapt to the object recognition task. The locomotion data were expressed as the number of crossings, while the exploration data were described as the number of rearings.

#### 5.4.2 Memory and Cognition Evaluation

The object recognition task was carried out on the 12th and 13th days of the experimental protocol, following the method previously described by Stangherlin et al.^70^ This behavioral test is commonly used to assess animals’ short-term memory (STM) and long-term memory (LTM).

After the habituation, the mice underwent a training session. Each mouse was placed individually in the testing area with two identical objects, A1 and A2, for 5 min. The exploration was recorded when the mouse directed its nose within approximately 2 cm of the object, engaging in activities such as sniffing, touching, or looking at it. To assess STM, 1.5 h after the training session, the mice were presented with a familiar object (A1) and a novel object (B). The exploration time was 5 min to measure learning and recognition memory.

Subsequently, LTM was evaluated 24 h after the training session. The mice were allowed to explore a familiar object (*A*_1_) and a novel object (*C*) for 5 min, and the time spent exploring each object was recorded. The objects used in the experiments were positioned symmetrically within the testing area. Objects *A*_1_ and *A*_2_ were identical balls; object *B* was a cube, and object *C* was a cylinder. These objects were made of plastic and measured 10 × 10 cm (length × height) with distinct color patterns (blue, red, and yellow). Between each trial, the arena and objects were cleaned with 30% ethanol to remove the residues and odors. The data obtained from the experiment were expressed as percentages of exploratory preference, calculated as follows

$$Training:\left(A_{2}/\left(A_{1}+A_{2}\right)\right)\times 100$$

$$STM:\left(B/\left(A_{1}+B\right)\right)\times 100$$

$$LTM:\left(C/\left(A_{1}+C\right)\right)\times 100$$

#### 5.4.3 Assessment of Depressive-like Behavior

The tail suspension test was conducted as described by Steru et al. at^71^ on day 14 of the experimental protocol. The mice were suspended 50 cm above the ground by an adhesive tape placed approximately 1 cm from the tip of the animal’s tail. Two parameters were evaluated during the behavioral test to assess the depressive-like response: the latency time (first episode of immobility) and the general immobility time. The test lasted 6 min in total. Mice were considered immobile only when passively hung and completely immobilized. Latency time and general immobility were recorded in seconds.

#### 5.4.4 Assessment of Anxious-like Behavior

On the 15th day of the experimental protocol, the elevated plus maze test (EPM) was performed to measure rodent anxiety-like behavior, following the procedure described by Pellow et al.^72^ The EPM apparatus consisted of two open arms (16 × 5 cm) and two closed arms (16 × 5 × 10 cm) arranged at a 90° angle, all connected to a central platform (5 × 5 cm) elevated 50 cm from the floor. Each animal was individually placed in the maze’s center, facing one of the open arms.

During a 5 min session, the frequency of entries into the open and closed arms, the time spent in the open arm, and the number of head dips (leaning toward the edge) were recorded. The data were expressed as a percentage of entries (using all four paws) and time spent in the open arms relative to the total number of entries and time spent in both open and closed arms, respectively. The number of entries into the closed arms was also documented.

### 5.5 Biochemical Assays

Biochemical tests were carried out to investigate the mechanisms altered by VCR that led to the development of depressive-like and anxiety-like behaviors as well as cognitive deficits. Here, the mechanistic action of VCR and 4-PSQ in relation to sex-dependent changes was observed to elucidate the possible predisposition to comorbidities among males and/or females.

We verified enzymatic damage to Na^+^, K^+^-ATPase, which can promote damage to neurochemical signaling and thus cause cognitive and emotional damage. The nervous system structures examined for oxidative damage and changes in Na^+^, K^+^ ATPase were the cerebral cortex, spinal cord, cerebellum, and hippocampus. Furthermore, the expression of NFκB and p21 investigated damage to the cell cycle and promotion of the neuroinflammatory response in the cerebral cortex and spinal cord. The other structures (cerebellum and hippocampus) were not used for gene expression analysis because the sample volume was very low and did not meet the sample RNA extraction minimal requirements (<50 mg).

#### 5.5.1 Tissue Preparation

On the day of euthanasia, samples of the cerebral cortex, spinal cord, cerebellum, and hippocampus were dissected. After separating the tissues, the samples were frozen and stored in an ultrafreezer (−80 °C). For the biochemical assays, the samples were homogenized in a 50 mM Tris–HCl buffer with pH 7.4 and centrifuged at 900*g* for 10 min to obtain the supernatant (S1).

Samples were prepared by using 50 mM Tris–HCl buffer (pH 7.4) at a 1:10 (w/v) ratio for ATPase activity analysis. For PCR assays, total mRNA was extracted from the tissue samples by using a preparation buffer containing Triton to ensure proper cell lysis and RNA preservation.

#### 5.5.2 Protein Quantification

Protein concentration was determined spectrophotometrically at 595 nm using Bradford method,^73^ with bovine serum albumin as a standard. The reaction mixture comprised S1 (50 μL) and Coomassie Brilliant Blue (2.5 mL). It was incubated for 10 min. The protein level was expressed in milligrams of protein per milliliter and used for various biochemical analyses.

#### 5.5.3 Na^+^, K^+^-ATPase Activity

To evaluate the activity of the Na^+^, K^+^ ATPase enzyme, the system was prepared with 3 mM MgCl_2_, 125 mM NaCl, 20 mM KCl, and 50 mM Tris/HCl (pH 7.4), along with an aliquot of S1, resulting in a final volume of 500 μL. The reaction was initiated by adding ATP to a final concentration of 3.0 mM. Control samples were prepared under the same conditions but with the addition of 0.1 mM ouabain, a Na^+^, K^+^ pump inhibitor. Reactions were started by adding adenosine triphosphate (ATP) and stopped after 30 min of incubation by adding 10% trichloroacetic acid (TCA). The samples were further incubated at 37 °C for 30 min, and the incubation was halted by adding 10% TCA with 10 mM HgCl_2_. Enzyme activity was calculated by measuring the difference in inorganic phosphate (Pi) levels between incubations performed in the absence and presence of ouabain.

The quantification of inorganic phosphate (Pi) released by ATPase enzymes was assessed using the method described by Fiske and Subbarow.^74^ Colorimetric reactions were analyzed spectrophotometrically at 650 nm. The results were expressed in nanomoles of Pi/mg protein/min.

#### 5.5.4 RNA Extraction and Quantitative RT-PCR

To evaluate the expression of p21 and NFκB, real-time RT-qPCR was performed. Total RNA was isolated from cerebral cortical and spinal cord clusters (*n* = 6) using TRIzol reagent (Invitrogen, Carlsbad, CA). The concentration and purity of the RNA were determined spectrophotometrically at a ratio of 260/280. Then, 1 μg of total RNA was reverse transcribed using the Applied Biosystems High Capacity cDNA Reverse Transcription Kit (Applied Biosystems, Foster City, CA) in a 20 μL reaction according to the manufacturer’s instructions. Quantitative PCR determination of the of the mRNAs (mRNAs) encoding p21 (#Rn00589996_m1), NFκB p65 (#Rn01502266_m1), and β-actin (#Rn00667869_m1) was performed using the TaqMan real-time RT-PCR system with inventory primers and probes purchased from Applied Biosystems (Thermo Fisher Scientific), as referred for each gene. Quantitative RT-PCR was performed in duplicate using an Applied Biosystems 7500 Fast system. Nontemplate controls were used in each assay, producing no detectable signal during 40 amplification cycles. Target mRNA levels were normalized to β-actin levels using the 2^–ΔΔCt^ method.^75^

### 5.6 Statistical Analysis

The normality of the data was assessed by using the Shapiro–Wilk normality test. Statistical analysis was conducted using GraphPad Prism 7.0 software (San Diego, CA, USA). No outliers were identified. Two-way ANOVA followed by Tukey’s multiple comparisons test was employed for statistical analysis. Data were presented as the mean ± S.E.M. Statistical significance was defined as *P* < 0.05.

Pearson’s correlation analysis was used to statistically verify the relationship between the development of depressive-like and anxious-like behaviors and cognitive deficit with changes in NFκB gene expression and Na^+^, K^+^-ATPase activity. The data chosen for statistical correlation analysis refer to the control and VCR groups of males and females. Values of *r* ≅ 1 indicating a positive correlation between the variables were represented by blue. Values of *r* ≅ −1, indicating a negative correlation between the two variables, are represented on the graph in red.

## Supplementary Material



## Notes

p-Values from Pearson’s correlation analyses between behavioral measures, number of entries into the open arms (anxiety-like behavior), exploratory preference (memory assessment), and immobility time (depressive-like behavior), and biochemical changes in the cerebral cortex of male mice; the same correlation analyses for female mice; and GC–MS and NMR data supporting the chemical/analytical characterization (PDF)

## Abbreviations

VCR: Vincristine
4-PSQ: 7-chloro-4-(phenylselanyl)­quinoline
NFκB: nuclear factor kappa B
p21: protein-21
JAK2: Janus Kinase 2
STM: short-term
LTM: long-term
CNS: central nervous system
EPM: elevated plus maze test
NO: nitric oxide
CDK: cyclin-dependent kinases
PI3K: phosphatidylinositol-3-kinase
AKT: protein kinase B
