# Supplementary material for: Correlation between NFκB Signaling and Na+, K+‑ATPase Inhibition in Vincristine-Induced Emotional and Cognitive Comorbidities in Mice: Neuroprotective Potential of 4‑PSQ
Source: ACS Omega. 2026 Feb 9;11(7):11219–32. doi: 10.1021/acsomega.5c07423 (PMC12947186; doi:10.1021/acsomega.5c07423)
Supplement: Supplementary file 1 [file ao5c07423_si_001.pdf]

Supplementary material

Correlation between NFκB Signaling and Na^+, K^+-ATPase Inhibition in Vincristine-Induced Emotional and Cognitive Comorbidities in Mice: Neuroprotective Potential of 4-PSQ

Ketlyn Pereira da Motta[a], Carolina Cristóvão Martins[a], Vanessa Macedo Esteves da Rocha[a], Ingrid Cardoso Oliveira[b], Diego Alves[b], Larissa Daniele Bobermin[c], André Quincozes-Santos[c], Ethel Antunes Wilhelm[a*]

Table S1. P-values from Pearson's correlation test between the number of entries into the open arms (anxious-like behavior), exploratory preference (memory assessment), and immobility time (depressive-like behavior) with biochemical changes in the cerebral cortex of male mice.

Experimental groups	***P-values***					
	Open arms entries	STM	LTM	Immobility	Na^+, K^+ - ATPase	NFκB
Open arms entries		2.46 $x10^{-4}$	0.001	0.032	0.013	0.062
STM	2.46 $x10^{-4}$		2.43 $x10^{-4}$	0.009	9.3$x10^{-4}$	0.059
LTM	0.001	2.43 $x10^{-4}$		0.021	0.005	0.034
Immobility	0.032	0.009	0.021		0.043	0.001
Na^+, K^+- ATPase	0.013	0.001	0.005	0.044		0.040
NFκB	0.062	0.059	0.034	0.001	0.040	

Abbreviations: Short-term memory (STM); Long-term memory (LTM); Nuclear factor kappa B (NFκB)

Table S2. P-values from Pearson's correlation test between the number of entries into the open arms (anxious-like behavior), exploratory preference (memory assessment), and immobility time (depressive-like behavior) with biochemical changes in the cerebral cortex of female mice.

Experimental groups	***P-values***					
	Open arms entries	STM	LTM	Immobility	Na^{+}, K^{+}- ATPase	NFκB
Open arms entries		0.002	0.004	3.27×10^{-4}	0.032	0.003
STM	0.002		0.009	3.75×10^{-5}	0.017	4.29×10^{-4}
LTM	0.005	0.010		0.001	2.34×10^{-5}	0.005
Immobility	3.27×10^{-4}	3.75×10^{-5}	0.001		0.003	0.001
Na^{+}, K^{+}- ATPase	0.033	0.017	2.00×10^{-5}	0.003		0.012
NFκB	0.003	4.29×10^{-4}	0.005	0.001	0.019	

Abbreviations: Short-term memory (STM); Long-term memory (LTM); Nuclear factor kappa B (NFκB)

GC-MS and NMR data

General Remarks: The progress of the reaction was monitored by thin-layer chromatography (TLC) on Merck silica gel 60 F254 plates, using UV light for visualization and a 5% vanillin solution in 10% H_2SO_4 followed by heating for spot development. Flash column chromatography was performed on Baker silica gel (particle size 0.040–0.063 mm). ^{1}H NMR spectra were recorded at 400 MHz on a Bruker Avance III HD spectrometer in $CDCl_3$ solutions. Chemical shifts (δ) are given in parts per million (ppm) relative to tetramethylsilane (TMS) as an internal standard, and coupling constants (J) are reported in Hertz (Hz). ^{13}C NMR spectra were obtained at 100 MHz on the same instrument, with chemical shifts reported in ppm relative to the residual solvent signal of $CDCl_3$. Low-resolution mass spectra (LRMS) were obtained on a Shimadzu GCMS-QP2010 mass spectrometer, and high-resolution mass spectra (HRMS) were recorded on a Bruker microTOF-QII spectrometer.

General Procedure for the Synthesis of 7-chloro-4-(phenylselanyl)quinoline: To a round-bottomed flask containing a solution of diphenyl diselenide (0.25 mmol) in PEG-400 (1.0 mL) under a nitrogen atmosphere was added $NaBH_4$ (1.0 mmol). The resulting mixture was stirred at 60 °C for 1 h, during which the color changed from yellow to colorless. Subsequently, 4,7-dichloroquinoline (0.5 mmol) was added, and the reaction was stirred at 60 °C for an additional 1 h. The reaction mixture was then poured into water (10 mL) and extracted with ethyl acetate (3 × 5 mL). The combined organic layers were dried over anhydrous $MgSO_4$ and concentrated under reduced pressure. The crude residue was purified by column chromatography on silica gel using ethyl acetate/hexane (20:80, v/v) as the eluent.

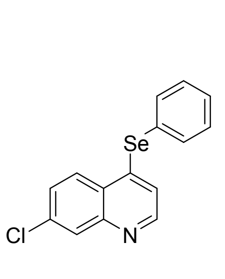

Spectral and analytical data for: 7-chloro-4-(phenylselanyl)quinoline: Yield: 0.156 g (98%); pale yellow solid; mp 81-83 °C. ^{1}H NMR (400 MHz, $CDCl_3$); *δ* (ppm): 8.51 (d, *J* = 4.7 Hz, 1H), 8.07 (d, *J* = 2.0 Hz, 1H), 7.99 (d, *J* = 8.9 Hz,

1H), 7.66-7.63 (m, 2H), 7.53-7.42 (m, 4H), 6.97 (d, J = 4.7 Hz, 1H). ^{13}C NMR (100 MHz, $CDCl_3$) δ = 150.27, 147.97, 145.89, 136.16, 135.44, 129.95, 129.34, 128.75, 127.39, 126.48, 126.07, 125.82, 121.70. MS (relative intensity) *m*/*z*: 321 (42), 320 (21), 319 (100), 317 (49), 284 (33), 282 (18), 241 (25), 239 (77), 204 (70), 162 (18), 142 (15), 135 (30), 127 (29), 99 (44), 77 (29), 75 (11), 51 (25). HRMS calcd. for $C_{15}H_{11}ClNSe$: $[M+H]^+$ 319.97452. Found: 319.97546.

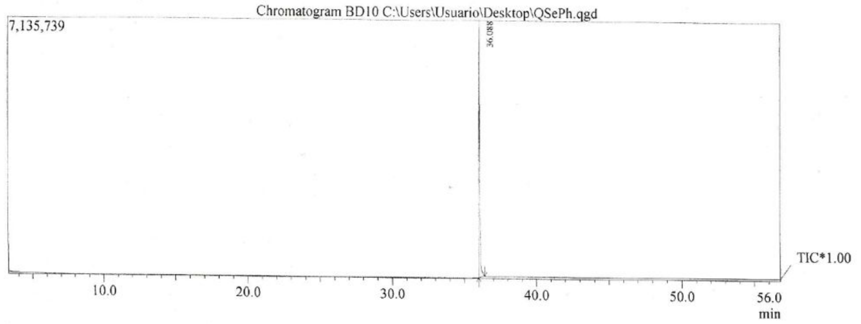

Peak Report TIC

Peak#	R.Time	I.Time	F.Time	Area	Area%	Height	Height%	A/H	Mark	Name
1	36.088	36.000	36.417	31558122	100.00	7099734	100.00	4.44	MI	
				31558122	100.00	7099734	100.00			

Spectrum

Line#:1 R.Time:36.1(Scan#:3934)
MassPeaks:44
RawMode:Averaged 36.1-36.1(3931-3935) BasePeak:319(537230)
BG Mode:Averaged 35.3-35.4(3839-3854) Group 1 - Event 1

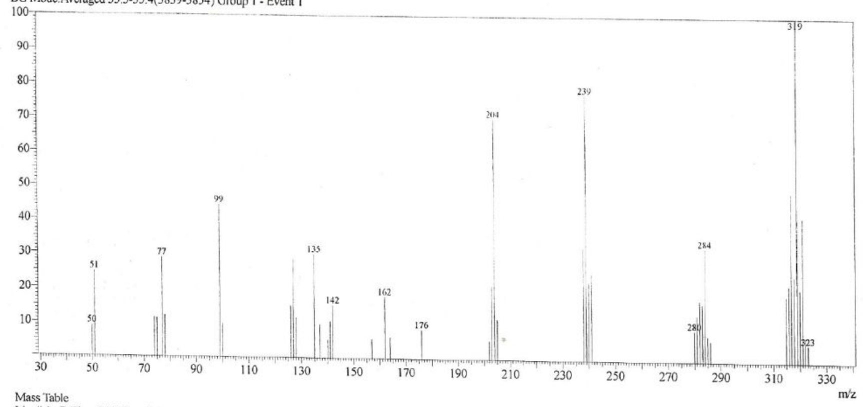

Mass Table
Line#:1 R.Time:36.1(Scan#:3934)
MassPeaks:44
RawMode:Averaged 36.1-36.1(3931-3935) BasePeak:319(537230)
BG Mode:Averaged 35.3-35.4(3839-3854) Group 1 - Event 1

#	m/z	Abs. Int.	Rel. Int.	#	m/z	Abs. Int.	Rel. Int.	#	m/z	Abs. Int.	Rel. Int.
1	49.95	48426	9.01	16	142.00	82399	15.34	31	281.95	95317	17.74
2	50.95	132734	24.71	17	156.95	30474	5.67	32	282.95	90351	16.82
3	73.95	61260	11.40	18	162.00	96238	17.91	33	284.00	176143	32.79
4	74.95	60576	11.28	19	164.00	33523	6.24	34	285.00	40508	7.54
5	77.00	154640	28.78	20	176.00	45906	8.54	35	286.00	32666	6.08
6	78.00	64916	12.08	21	202.00	29908	5.57	36	314.95	102864	19.15
7	99.00	238523	44.40	22	203.00	115437	21.49	37	315.95	120015	22.34
8	100.00	52541	9.78	23	204.05	377342	70.24	38	316.95	264033	49.15
9	126.05	81842	15.23	24	205.05	64363	11.98	39	317.95	133968	24.94
10	127.05	154740	28.80	25	238.00	175874	32.74	40	319.00	537230	100.00
11	128.00	63756	11.87	26	239.00	415396	77.32	41	320.00	113887	21.20
12	135.00	161646	30.09	27	240.00	122724	22.84	42	321.00	224889	41.86
13	137.00	52471	9.77	28	241.00	135141	25.16	43	321.95	40775	7.59
14	140.05	28564	5.32	29	279.95	48817	9.09	44	322.95	27830	5.18
15	141.00	58010	10.80	30	280.95	73324	13.65				

Figure S1. Chromatogram and Mass Spectrum for **7-chloro-4-(phenylselanyl)quinoline**.

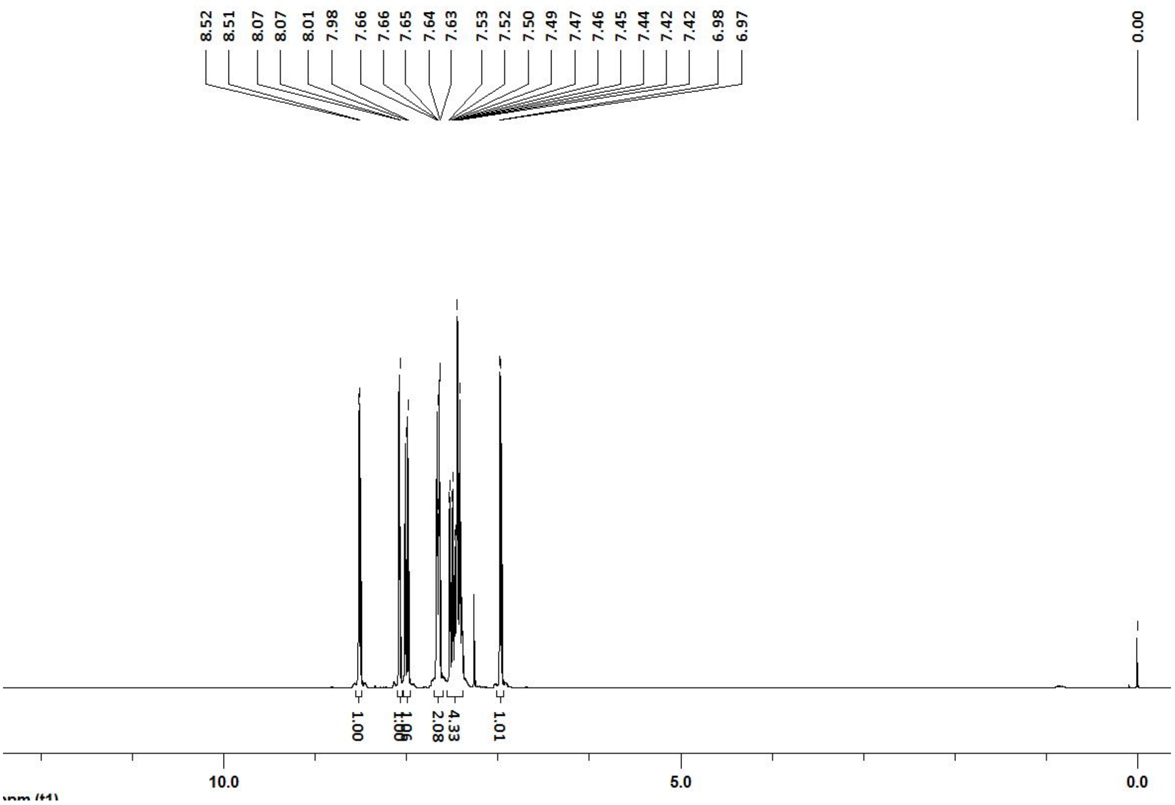

Figure S2. ^{1}H NMR (400 MHz) spectrum for **7-chloro-4-(phenylselanyl)quinoline** in $CDCl_3$.

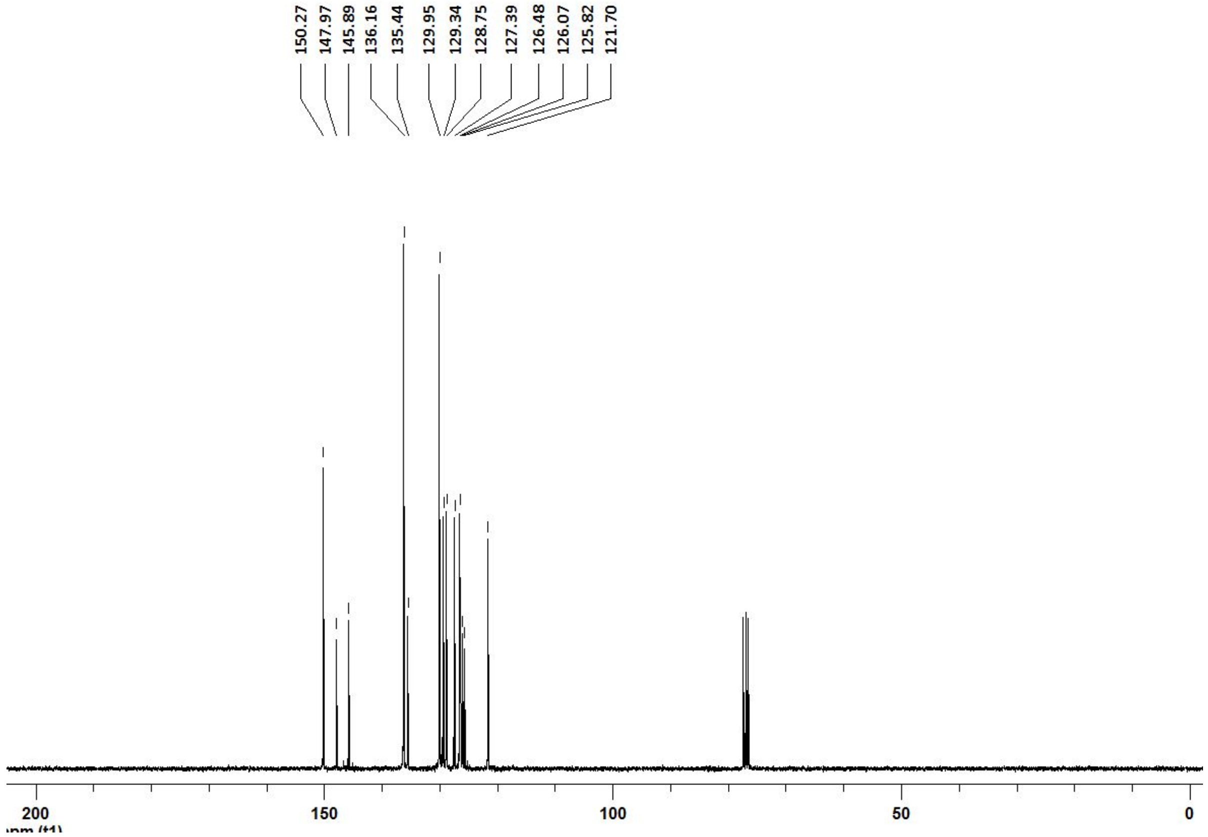

Figure S3. ^{13}C NMR (100 MHz) spectrum for **7-chloro-4-(phenylselanyl)quinoline** in $CDCl_3$.

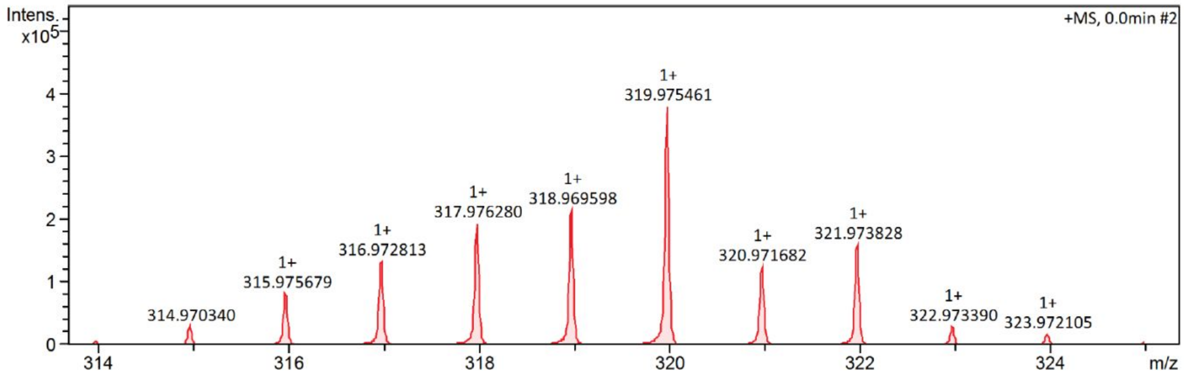

Figure S4. HRMS spectrum for **7-chloro-4-(phenylselanyl)quinoline**.
